# Dynamic contrast-enhanced photoacoustic imaging using photothermal stimuli-responsive composite nanomodulators

**DOI:** 10.1038/ncomms15782

**Published:** 2017-06-08

**Authors:** Yun-Sheng Chen, Soon Joon Yoon, Wolfgang Frey, Mary Dockery, Stanislav Emelianov

**Affiliations:** 1 Department of Radiology, Stanford University, 300 Pasteur Drive, Stanford, California 94305–5105, USA; 2 Department of Electrical and Computer Engineering, The University of Texas at Austin, 1 University Station, Austin, Texas 78712, USA; 3 Department of Biomedical Engineering, The University of Texas at Austin, 1 University Station, Austin, Texas 78712, USA; 4 School of Electrical and Computer Engineering, Georgia Institute of Technology, 777 Atlantic Drive, Atlanta, Georgia 30332, USA; 5 Wallace H. Coulter Department of Biomedical Engineering, Georgia Institute of Technology and Emory University School of Medicine, 313 Ferst Drive, Atlanta, Georgia 30332, USA

## Abstract

Molecular photoacoustic imaging has shown great potential in medical applications; its sensitivity is normally in pico-to-micro-molar range, dependent on exogenous imaging agents. However, tissue can produce strong background signals, which mask the signals from the imaging agents, resulting in orders of magnitude sensitivity reduction. As such, an elaborate spectral scan is often required to spectrally un-mix the unwanted background signals. Here we show a new single-wavelength photoacoustic dynamic contrast-enhanced imaging technique by employing a stimuli-responsive contrast agent. Our technique can eliminate intrinsic background noises without significant hardware or computational resources. We show that this new contrast agent can generate up to 30 times stronger photoacoustic signals than the concentration-matched inorganic nanoparticle counterparts. By dynamically modulating signals from the contrast agents with an external near-infrared optical stimulus, we can further suppress the background signals leading to an additional increase of more than five-fold in imaging contrast *in vivo*.

## Abstract (web summary)

Photoacoustic imaging becomes an enabling technology that is designed for clinic diagnosis of disease. Here, Chen *et al*. report an imaging contrast agent—plasmonic nanoparticles caged in hydrogel subject to reversible volume change depending on temperature, which exhibits tunable photoacoustic signal.

In molecular photoacoustic imaging, the imaging contrast is associated with the difference in optical absorption between that of the imaging contrast agent and that of the background tissue. Some types of tissue produce strong endogenous background signals that can generate false positive results in contrast-enhanced photoacoustic imaging. To rule out these false positives, a spectral scan, that sweeps the illuminating laser light across multiple wavelengths, is often needed. These spectral scans can differentiate molecular contrast agents from tissues based on their unique spectral characteristics. However, spectral scanning reduces imaging speed and introduces complexities in the un-mixing process because of the wavelength-dependent optical absorption and scattering of tissue. Furthermore, the signals from endogenous chromophores are much higher than the signals from the contrast agents, thus a higher concentration of contrast agents is often required, leading to a potential concern of long-term cytotoxicity.

Dynamic contrast-enhanced imaging aims to visualize targets that generate lower signals than their surrounding background tissue^1^. On the basis of different dynamic responses to an external modulated stimulus, the imaging target generates signals that follow the modulation frequency of the stimulus, while the background generates a static or randomly fluctuating signal. The dynamic signals from the target can be tracked over time and separated from the background. However, only a few photoacoustic imaging agents can respond to external stimulus repeatedly^2^,^3^,^4^,^5^. Most of these stimuli-responsive agents are activatable fluorescent dyes or proteins^2^,^3^, a stimulus-responsive fluorophore that can change its optical absorption in response to a stimulus signal. These imaging agents have been widely used in fluorescent imaging and partially adopted for photoacoustic imaging^2^,^6^,^7^,^8^,^9^,^10^. However, such fluorophore-based contrast agents are relatively inefficient and less thermodynamically stable than colloidal nanoparticles, leading to a lower (micro-molar) imaging sensitivity. Also, most of these agents require an optical stimulus in a short wavelength (<800 nm) and none can currently operate in the wavelength range within the second near infrared window of tissue (1–1.4 μm), a wavelength range in which tissue generates the weakest background photoacoustic signals. Furthermore, although small molecular fluorophores and genetic encoded fluorescent proteins provide many superior advantages in imaging diagnosis, they are less ideal for theranostic applications, where the imaging contrast agents can also function as or carry therapeutic agents.

Here, we develop first stimuli-responsive photoacoustic contrast agents that work in both first (650–950 nm) and second (1,000–1,700 nm) near infrared optical windows of tissue. Using such contrast agents, we also demonstrate a new approach of dynamic contrast-enhanced photoacoustic imaging *in vivo*. It is well known that a nanoparticle solution, unlike a homogeneous medium, is optically and thermally heterogeneous with a photothermal excitation. Because of the small size of nanoparticles, thermal energy generated from optical absorption diffuses out rapidly into the surrounding medium, and the intensity of photoacoustic signal is proportional to this outgoing thermal flux^11^. As a result of heat dissipation, photoacoustic intensity of tightly aggregated nanoparticle increases significantly as the heating volumes of each single particle overlap, which increases the thermal gradient of the cluster^12^. We thus hypothesize that a nanoparticle cluster, in which the inter-particle separation can be varied in a controlled manner, can be an efficient stimuli-responsive probe for dynamic contrast-enhanced photoacoustic imaging.

## Results

### Development of the stimuli-responsive contrast agents

To test our hypothesis, we develop a new nanocomposite contrast agent, composed of a photothermal stimuli-responsive hydrogel nanocage loaded with inorganic nanoparticles that are commonly used photoacoustic contrast agents with absorption peaks in the near infrared wavelength range. In this design, the switchable photoacoustic signal comes from reversible nanoparticle aggregation. Numerous stimuli-responsive hydrogel nanoparticles (nanogels), initially designed for controlled drug releasing, are excellent platforms for this application because they can recognize stimuli such as specific chemicals, pH value, temperature or light and undergo a volumetric change^13^,^14^. Here, we choose a volume-changing photothermal stimuli-responsive nanogel, poly(n-isopropylacrylamide) (PNIPAM), to carry nanoparticles and control their aggregation^13^,^15^.

PNIPAM-based nanogels have been widely used for tissue engineering, tunable catalysis, sensing^16^, controlled release of therapeutic agents such as genes and drugs^17^,^18^,^19^, and optical imaging^20^. To boost photoacoustic intensity, PNIPAM nanogels are advantageous as their volume rapidly reduces (that is, de-swelling state or shrink state) as temperature rises above their lower critical solution temperature (LCST), a phenomenon that is caused by phase transition of hydrophobic moieties of the polymer chain^15^. The LCST of PNIPAM is 32 °C, but can be adjusted to match the biological temperature (37 °C) by co-polymerizing hydrophilic monomers such as acrylic acid and acrylamide into the polymer matrix^21^,^22^,^23^. Importantly, the phase-transition process is reversible: as temperature decreases, the nanogels expand and resume their initial volumes (that is, swelling state).

We synthesize PNIPAM nanogels loaded with nanoparticles (PNIPAM-nanoparticle nanoconstructs, see Methods). Two classes of nanoparticles commonly used as photoacoustic contrast agents, gold nanorods (AuNRs) and copper sulfide nanospheres (CuS NSs), are chosen as examples. Each class of nanoparticles is separately loaded into PNIPAM nanogels to form two types of stimuli-responsive photoacoustic contrast agents (PNIPAM-AuNR and PNIPAM-CuS nanoconstructs)^24^,^25^,^26^,^27^. Gold nanorods have a large optical cross-section, tunable absorption peaks and inert chemical reactivity. However, due to plasmonic coupling, optical absorption of AuNRs changes when they are brought close to each other. To demonstrate that the signal boost in our dynamic contrast agents is attributed to the thermo-dynamic effect, we load CuS NSs into PNIPAM nanogels: although the optical absorption cross-section of CuS NSs is more than one order of magnitude lower than that of AuNRs and their absorption peak is less tunable, optical absorption of CuS NSs does not change with nanoparticle aggregation. In addition, their much smaller dimensions and inherent absorption peak within the second optical window of tissue makes them attractive photoacoustic contrast agents^28^.

### Characterization of the nanoconstructs

**Figure 1:**
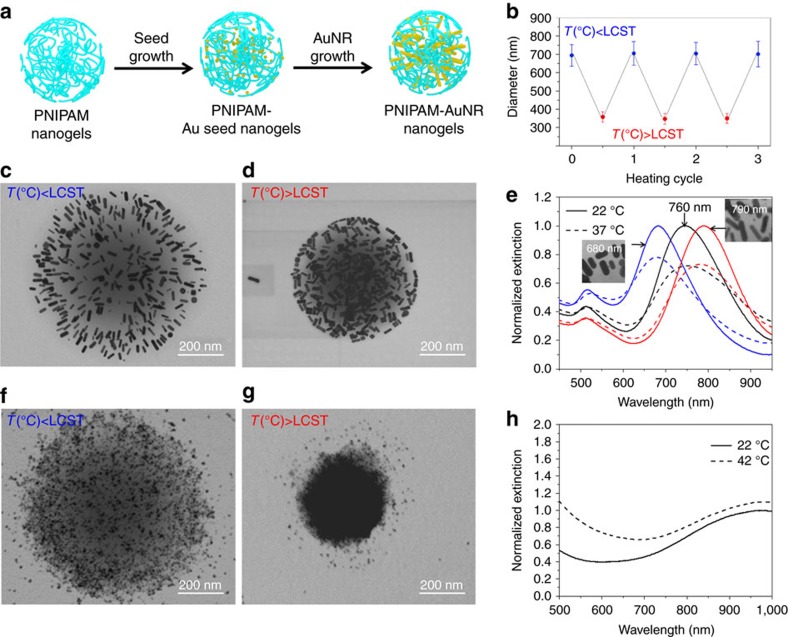
Synthesis and characterization of PNIPAM-nanoparticle nanoconstructs. (**a**) Schematic of the PNIPAM-AuNR synthesis. (**b**) Averaged diameter and s.d. of PNIPAM below and above LCST over 3 heating and cooling cycles measured by DLS. The error bars represent the s.d. of the size distribution. (**c**) Transmission electron microscope (TEM) images of PNIPAM-AuNR nanoconstructs prepared at temperature below LCST. (**d**) TEM images of PNIPAM-AuNR nanoconstructs prepared at temperature above LCST. (**e**) UV-Vis spectroscopy of PNIPAM-AuNR with various resonance peaks by changing the nanorod aspect ratios. UV-Vis spectra at 22 °C (solid curves) and 37 °C (dotted curves) are recorded, featuring the temperature below and above LCST of PNIPAM. (**f**) TEM images of PNIPAM-CuS nanoconstructs prepared at temperature below LCST. (**g**) TEM images of PNIPAM-CuS nanoconstructs prepared at temperature above LCST. (**h**) UV-Vis spectra of PNIPAM-CuS at 22 °C (solid curves) and 42 °C (dotted curves).

Both nanoconstructs have an average diameter of 700 nm (±70 nm) at room temperature (Fig. 1). After heating above the LCST, the 700 nm nanogels shrink to 350 nm (±33 nm), corresponding to 12.5% of their original volume (Fig. 1b). To investigate morphological changes of these nanoconstructs induced by thermal stimulation, we acquire transmission electron microscopy (TEM) images of the samples at temperatures below and above the LCST. TEM images (Fig. 1c,d,f,g) at different temperatures illustrate the swelling and de-swelling states of these nanoconstructs. The PNIPAM-AuNR nanoconstructs show that AuNRs are more likely to orient along the radial direction (Fig. 1c) once they are at the swelling state; but above the LCST at the de-swelling state, the nanorods are more prone to align along the tangential direction (Fig. 1d). The change of nanorod orientations in self-assembly can be attributed to minimizing the surface energy of the rods in each PNIPAM morphology state.

Above the LCST, the volume reduction of the nanoconstructs also results in changes of their optical properties. Optical absorption of PNIPAM-AuNRs is determined by two dominant factors: localized surface plasmon resonance (LSPR) of a single AuNR, and plasmonic and thermal coupling between AuNRs. The LSPR of a AuNR is determined by its geometry or, specifically, aspect ratio, which can be readily tuned during the synthesis (Fig. 1e). The plasmonic coupling, however, is strongly affected by spatial arrangements of the nanorods, such as separation distances and orientations. The change of the AuNR re-orientation and reduced separation result in stronger coupling and shift of plasmonic resonance. Indeed, the ultraviolet–visible (UV-Vis) spectra (Fig. 1e) show that the phase transition causes 40–50% broadening of the full width at half maximum (FWHM), 20–30% reduction of the extinction, and a 2–8 nm red shift of the absorption peak. This reduced optical absorption also reconfirms our assumption that the enhanced photoacoustic signals are purely attributed to a thermal dynamic effect rather than the optical absorption of the particles.

In contrast to AuNRs, CuS NSs are semiconductor nanoparticles; their optical absorption is caused by electronic transition overcoming their bandgap^27^. As CuS NSs are brought close to each other (Fig. 1f,g), the increase in their extinction at short wavelengths comes mainly from the increased Rayleigh scattering of the nanoconstructs due to the aggregation (Fig. 1h).

### Photoacoustic signal characterization using phantoms

**Figure 2:**
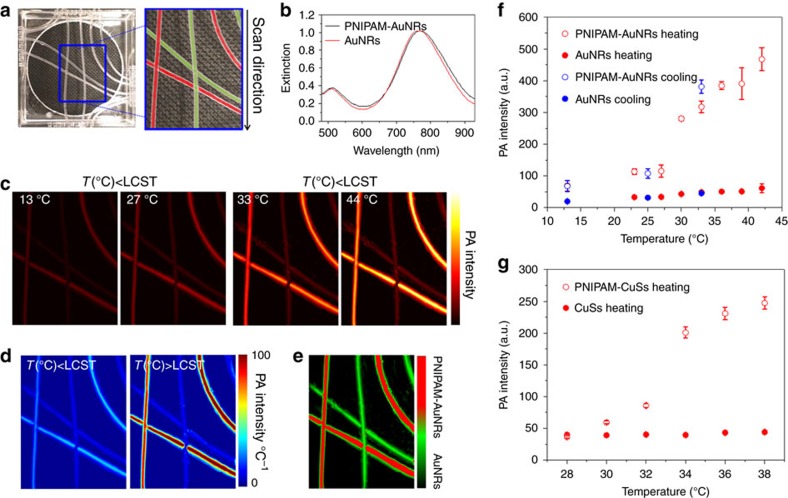
Photoacoustic imaging of nanoconstructs at temperatures from 13 to 44 °C. (**a**) Photograph (left) of thin wall polyethylene tubes filled with PNIPAM-AuNR and pure AuNR solutions. The tube generates negligible photoacoustic signals. Photograph (right) is colour-coded: red indicates PNIPAM-AuNR, green indicates pure AuNR. (**b**) UV-Vis or Ultraviolet-visible extinction spectra indicate the matched extinction of AuNRs in both the nanoconstructs and the pure AuNR solutions at 760 nm. (**c**) Photoacoustic imaging of the tissue-mimicking phantom at temperatures 13, 27, 33 and 44 °C (two temperatures below LCST, and two above LCST). (**d**) Photoacoustic intensity slope images below and above LCST. The colour map shows the photoacoustic intensity change per change of temperature. Comparing the two imaging, it shows the slope of pure AuNR stays unchanged below and above LCST, whereas the slope of photoacoustic intensity from PNIPAM-AuNR changes dramatically. (**e**) Using the difference photoacoustic slopes from (**d**) to differentiate the PNIPAM-AuNR from AuNRs. (**f**) Comparison of photoacoustic intensities for PNIPAM-AuNR and pure AuNR with a heating and cooling cycle, indicating the curve retrieves back to its original photoacoustic intensity as temperature cools below LCST. Abrupt photoacoustic intensity changes are observed in both PNIMPAM-AuNR nanoconstructs and (**g**) PNIPAM- CuS nanoconstructs, whereas their pure nanoparticles (AuNR and CuS) show linear behaviour with respect to temperatures. For a fair comparison of the signals, the signal calibration in (**f**,**g**) is based on a phantom containing two parallel tubes. One tube contains the nanoconstructs and the other one contains pure AuNR or CuS nanopartilces with matched concentration. The positions of these two tubes are carefully leveled with ultrasound imaging; the difference of their positions relative to the transducer is <1 mm. The overall intensity is the integrated photoacoustic signal inside the whole tube in the field of view defined by ultrasound image. The concentration of the particle solutions are matched to OD=3.33 for 1 cm optical path in these measurements. The error bars in (**f**,**g**) represent the s.e. of mean of 300 frames of photoacoustic images.

As predicted by the theoretical analysis (Supplementary Figs 1,2, Supplementary Note 1), the morphological change of the nanoconstructs should induce different photoacoustic responses. To demonstrate this effect, we perform photoacoustic imaging of PNIPAM-nanoparticle nanoconstructs using a photoacoustic imaging system and a tissue-mimicking phantom (Fig. 2a). The temperature of the phantom is controlled by a circulating water bath with a precision of 0.1 °C. Photoacoustic intensities from pure AuNR and PNIPAM-AuNR solutions are compared. AuNR concentrations are first matched to the same optical density (OD) through extinction spectroscopy for a fair comparison (OD=3.33 for 1 cm optical path, Fig. 2b). We record photoacoustic images as a function of the solution temperature between 13 and 44 °C (Fig. 2c). Our results show that the PNIPAM-AuNR solution produces 3.5 times higher photoacoustic intensity than the pure AuNR solution, even below the LCST (Fig. 2f). This photoacoustic signal enhancement is attributed to the randomly occurring overlaps of the thermal profiles that create a higher temperature gradient. Also in this temperature range, the photoacoustic intensity increases linearly with temperature in both solutions. This increment comes from the thermal expansion coefficient of water that increases linearly from 4 to 50 °C (ref. 11). Once the temperature exceeds the LCST, the pure AuNR solution maintains its linear photoacoustic intensity trend, whereas the PNIPAM-AuNR solution displays an abrupt additional intensity enhancement of 2.5 times (Fig. 2f). This increment happens as temperature transitions from 27 to 32 °C, near the LCST of PNIPAM, confirming our theoretical prediction that on phase transition of PNIPAM the sudden density increase of the loaded AuNRs (or reduced particle spacing) causes the photoacoustic signal enhancement. Above the LCST temperature range (from 32 to 44 °C), the photoacoustic intensity from PNIPAM-AuNR increases linearly with temperature again, but with a steeper slope. As a result, its photoacoustic enhancement factor increases by more than seven times compared with pure AuNR solution at 37 °C. Therefore, there are two types of photoacoustic intensity enhancements. The first, caused by nanoparticle clustering, is termed clustering enhancement. The second, caused by nanogel de-swelling, is termed Hoberman enhancement, where the nanoconstruct shrinks above LCST as the Hoberman sphere collapses into a smaller sphere. The Hoberman enhancement factor is defined as the ratio of the clustering enhancements between the de-swelling and swelling states. These results presented in Fig. 2 suggest that the developed PNIPAM-AuNR nanoconstruct is a more efficient photoacoustic emitter than AuNRs alone and, more importantly, can serve as a stimuli-responsive photoacoustic signal modulator. In addition, the unique photoacoustic signature from the Hoberman enhancement can be used to monitor the morphological changes of the nanoconstructs.

Once the temperature is cooled down below the LCST, the reversibility of this nanoconstruct shrinking process allows the photoacoustic intensity to reduce back to its original value by following the same curve, which describes the dependence of the signal with temperature (Fig. 2f). Because of its nonlinear temperature-dependent behaviour, PNIPAM-AuNR nanoconstructs can be distinguished among other strong light absorbers or unwanted static background noises with a single-wavelength laser illumination (Fig. 2d,e).

Similar photoacoustic signal characterization is performed using PNIPAM-CuS nanoconstructs and CuS NSs alone. The spherical nature of CuS NSs provides PNIPAM-CuS with a better photothermal stability than PNIPAM-AuNR, which can contribute to an improved repeatability as reusable stimuli-responsive agents. Although its trend in photoacoustic intensity increase is similar to that of PNIPAM-AuNR, the enhancement factor of PNIPAM-CuS is slightly different: 2 and 5.5 times clustering enhancements are observed at background temperatures lower and higher than the LCST, respectively (Fig. 2g). This difference can be attributed to the inherently lower absorption cross-section of the CuS NSs, which require the nano particles to pack more densely to achieve a comparable signal enhancement.

**Figure 3:**
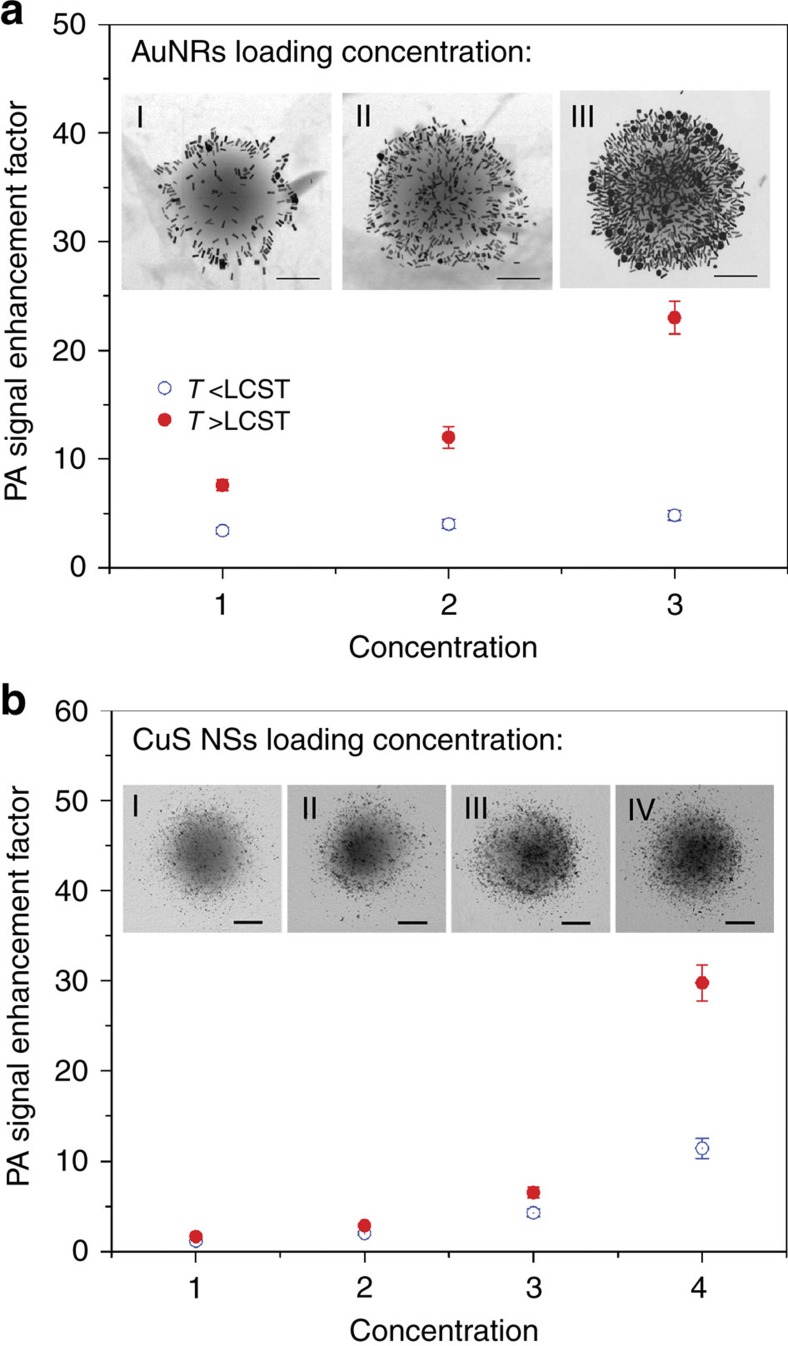
Enhancement factors as a function of loading concentrations of nanoparticles. (**a**) Photoacoustic signal enhancement factor as a function of AuNR loading concentration below (blue points) and above LCST (red points). Insets show TEM images of the nanoconstructs with increased loading density of AuNRs. (**b**) Photoacoustic enhancement factor increases as CuS nanosphere loading concentration increases. Four loading concentrations are tested. Insets show TEM images of PNIPAM-CuS nanoconstructs with increased loading density of CuS. The concentration of both PNIPAM-AuNRs and PNIPAM-CuSs are matched to the OD=3.33 (for 1 cm optical path) by UV-Vis spectroscopy for equal comparison. The error bars represent the s.e. of mean of 300 frames of photoacoustic images. Scale bars are 250 nm.

To investigate whether photoacoustic signal enhancement can be further improved, we prepare nanoconstructs with higher concentrations of nanoparticle loading. As shown in the TEM images in Fig. 3a, the AuNR concentration is varied by 2.5 and 6.2 times. From Fig. 3a III, in comparison with the same concentration of the pure AuNR solution, the PNIPAM-AuNR solution with the highest loading concentration reveals 5.4 and 22 fold clustering enhancements of photoacoustic signal at background temperatures lower and higher than the LCST, respectively. The smaller dimension of CuS NSs allows for a much higher loading concentration into a single PNIPAM nanogel. As a result, we expect a higher photoacoustic intensity increment from the densely loaded PNIPAM-CuS nanoconstructs. We prepare four different loading concentrations of both PNIPAM-CuS and CuS NSs, and test their clustering photoacoustic signal enhancement factors at 27 and 37 °C. Clustering enhancements of 11.5 and 30 times below and above the LCST are observed in the case of the highest loading concentration (Fig. 3b (IV)).

### *Ex vivo* dynamic contrast-enhanced photoacoustic imaging

**Figure 4:**
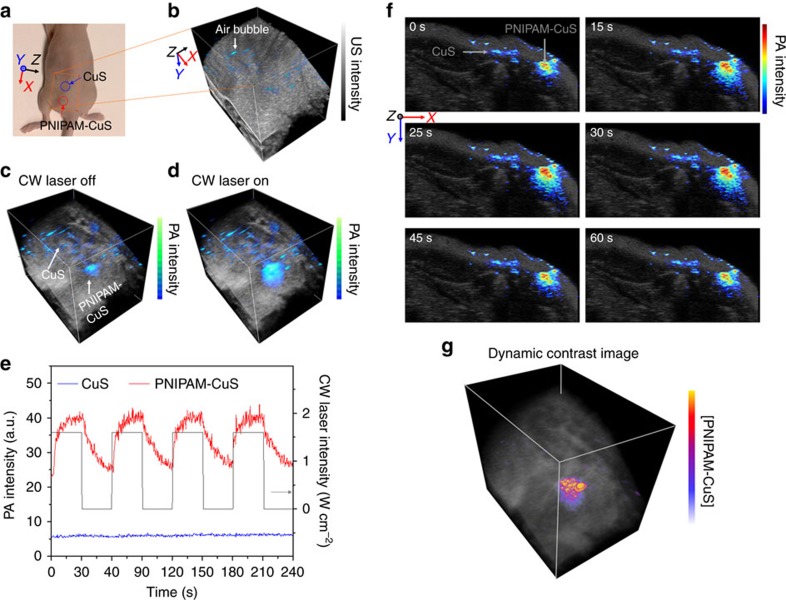
*Ex vivo* photoacoustic imaging of a mouse. (**a**) An image of a mouse model with both CuS NSs and PNIPAM-CuS injected in the region marked by the circles. (**b**) Overlay of ultrasound and photoacoustic images show scattered air bubbles on the skin as a strong background noise before nanoparticle injection. (**c**) After nanoparticle injection, when the CW laser is off, the region with PNIPAM-CuS is clearly visualized, but the region with CuS NSs is relatively weak, and difficult to be distinguished from the bubble region. (**d**) When the CW laser is on, the photoacoustic intensity at the PNIPAM-CuS region is dramatically enhanced. (**e**) Multi-cycles of CW laser on and off shows the photoacoustic signals follow the CW laser on/off cycle dynamically. (**f**) Photoacoustic intensity recorded during a full cycle of CW laser on and off. (**g**) Dynamic contrast-enhanced photoacoustic image obtained by subtracting the photoacoustic intensity under CW laser-off from CW laser-on, removing the strong background noises from the tissues, CuS NPs and bubbles and unambiguously reveals the region with only the PNIPAM-CuS. The image volume in (**b**–**d**,**g**) is 23 × 20 × 25.8 mm^3^.

It is known that under continuous wave (CW) laser illumination, optically absorbing nanoparticles will increase temperature rapidly^29^. This photothermal effect has been used for localized heating to destroy cancer cells^30^ and to trigger drug release^31^,^32^,^33^. It suggests that our PNIPAM-nanoparticle nanoconstructs can be noninvasively turned on and off with external lasers, a heating source that is simple to implement and can be controlled precisely. We test the photoacoustic responses of PNIPAM-CuS in an *ex vivo* animal model (Fig. 4a), and compare the results to those of CuS NSs with a matched concentration. Ultrasound and photoacoustic images are recorded and overlaid to show both anatomy of mouse tissue and the location of the particles (Fig. 4b). As expected, the area containing nanoconstructs produces a much stronger photoacoustic intensity (Fig. 4c).

To test the dynamic contrast behaviour of the nanoconstructs, the CW laser is periodically turned on and off. As shown in Fig. 4d, when the CW laser is on, in the region containing nanoconstructs, CuS NSs instantaneously heat up their PNIPAM cage and cause a noticeable increase of photoacoustic signal (Hoberman enhancement), whereas a minor photoacoustic intensity change is observed elsewhere (see Supplementary Movie 1 for a real-time record of photoacoustic images with repeated on-off cycling of the CW laser). This effect is quantified and plotted (Fig. 4e) to confirm that the repeated Hoberman enhancement of photoacoustic signals from the region containing nanoconstructs adiabatically follow the periodic laser heating, a unique signature that can also be used to differentiate the PNIPAM-CuS nanoconstructs from the tissue. In this experiment, to demonstrate the ability of visualizing the enhanced contrast, we intentionally introduce several small air bubbles on the mouse skin producing strong background noise (Fig. 4b). Because the background noise (tissue and bubbles) changes very little when the CW laser is on and off, by subtracting the photoacoustic images before and after CW heating, the signals from the background region and region with pure CuS NSs are suppressed, revealing only the regions with the nanoconstructs (Fig. 4f). This background suppression leads to about seven fold increase in the imaging contrast (see Methods, Data and statistical analysis section).

### *In vivo* dynamic contrast-enhanced photoacoustic imaging

For *in vivo* imaging studies, we prepare nanoconstructs with LCST higher than the normal physiological temperature of mice. A higher LCST of the nanogel can be achieved by co-polymerizing acrylamide to PNIPAM with a carefully adjusted molar ratio between n-isopropylacrylamide and acrylamide^23^. Poly(n-isopropylacrylamide-acrylamide) (PNIPAM-AM) is chosen because of its relatively faster de-swelling kinetics comparing to other high LCST PNIPAM derivatives or co-polymers^34^. The developed PNIPAM-AM nanogels are then coated with a thin layer (∼10 nm) of polyethylene glycol (PEG) to prolong the circulation^35^. We again grow AuNRs *in situ* within the LCST-adjusted PNIPAM-AM nanogels. Compared with PNIPAM, PNIPAM-AM has a slightly broader LCST. Temperature controlled dynamic light scattering (DLS) measurements indicate the average size of these nanoconstructs is about 350 nm (±234 nm) at room temperature, 320 nm (±175 nm) at 37 °C and 195 nm (±120 nm) at 44 °C. The swelling and de-swelling processes are repeatable (Supplementary Fig. 3). The UV-Vis spectrum of PNIPAM-AM-AuNR nanoconstructs shows similar optical properties as PNIPAM-AuNR nanoconstructs in both swelling and de-swelling states (Supplementary Fig. 4). Although DLS data of these nanoconstructs indicate a slight size reduction at 37 °C, their UV-Vis spectra do not show an observable increment of scattering, which indicates little aggregation at this temperature. Compared with the UV-Vis spectrum of PNIPAM-AuNRs above LCST (Fig. 1e, dotted line), the spectrum of PNIPAM-AM-AuNRs at 44 °C (Supplementary Fig. 4, red line) shows a relatively small change in scattering and peak broadening induced by plasmonic coupling. This difference comes from the relatively smaller size of the nanogels and consequentially lower loading quantities of AuNRs.

To confirm the delivery of PNIPAM-AM-AuNR nanoconstructs *in vivo*, we conjugate indocyanine green (ICG) dyes on the AuNR surface. The fluorescent signals from the dye allow us to simultaneously confirm the distribution of the nanoconstructs dynamically with fluorescent imaging. In the *in vivo* study, a mouse model of a prostate cancer is used where 100 μl of mixture of 5 × 10^6^ LNCaP cells and growth factor reduced Matrigel (1:1 volume ratio) is subcutaneously injected into the right flank of each mouse. Once tumour is developed, the tumour bearing mouse is intravenously injected 50 μl of nanoconstructs/phosphate-buffered saline solution (3 × 10^10^ nanoconstructs with ∼1 × 10^12^ AuNRs, OD=20 at 1 cm optical path). The distribution of the nanoconstructs is first visualized via epi-fluorescent imaging (IVIS Spectrum, PerkinElmer). The images (Supplementary Fig. 5) show the nanoconstructs disperse throughout the mouse immediately after the injection and start to clear out within one hour. We acquire dynamic contrast-enhanced photoacoustic imaging using the same method described in the *ex vivo* study. Briefly, photoacoustic images before and one hour after injection are acquired; the images indicate that the nanoconstructs indeed enhance the photoacoustic contrast (Supplementary Fig. 6c,d). However, due to the high concentration of nanoconstructs still circulating in the blood, the CW laser heating increases the photoacoustic intensity in both blood vessels and the tumour (Supplementary Fig. 6e), resulting in a dynamic contrast-enhanced image quite similar to the original photoacoustic image (Supplementary Fig. 6e).

**Figure 5:**
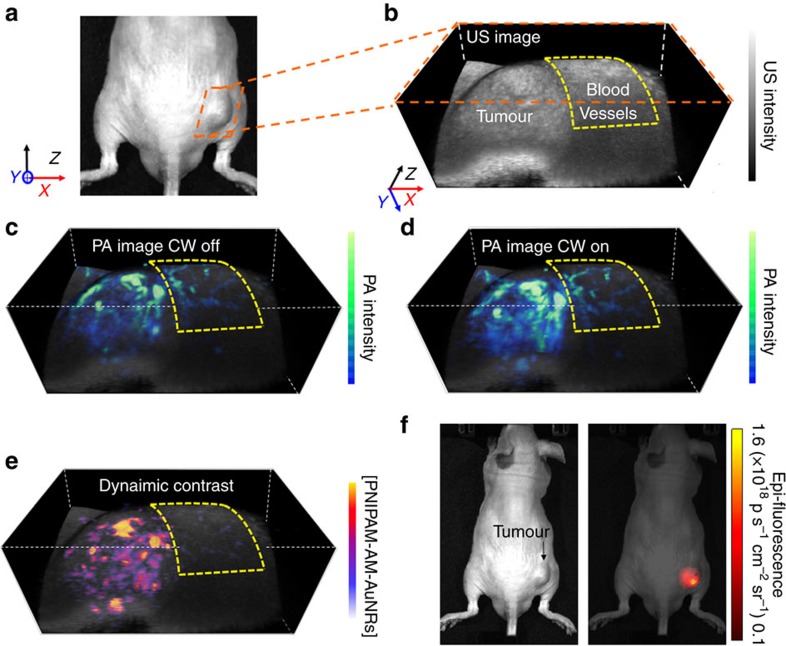
*In vivo* photoacoustic imaging of a tumour-bearing mouse. (**a**) An image of a subcutaneous prostate tumour in the right thigh of a mouse. (**b**) Ultrasound image of the tumour for image co-registration. (**c**) Photoacoustic image 24 h after nanoparticle injection shows the tumour, and blood vessels near the tumour (yellow box). (**d**) When the CW laser is on, the photoacoustic signal/image intensity from tumour is enhanced due to the de-swelling of the nanoconstructs, where the photoacoustic intensity of blood vessels barely increases. (**e**) Dynamic contrast-enhanced photoacousitc image obtained by subtracting the photoacoustic signals with CW laser off from photoacoustic signals with CW laser on, removing the photoacoustic signals from blood and background thus revealing the region with only the PNIPAM-AM-AuNRs. The image volume in (**b**–**e**) is 17.2 × 7.4 × 10.2 mm^3^. (**f**) *In vivo* epi-fluorescent imaging 24 h after the tail-vein injection visualizes the fluorescent signals of the indocyanine green (ICG) dyes conjugated with PNIPAM-AM-AuNR nanoconstructs.

However, 24 h after the injection, once most of the nanoconstructs clear from blood vessels, and accumulate in the tumour via enhanced permeability and retention (EPR) effect, the nanoconstructs are undetectable from blood vessels with the IVIS system (Fig. 5f and Supplementary Fig. 5e). Dynamic contrast-enhanced photoacoustic image highlights the suppression of the background signals from the blood vessels (shown in the yellow dotted box in Fig. 5c,d). As there are much lower amount of circulating nanoconstructs in the blood after 24 h of injection, comparing the photoacoustic images before and after the CW laser stimuli, only the tumour region, where the nanoconstructs accumulate, shows photoacoustic enhancements (Fig. 5d). By simply subtracting these static photoacoustic images, the signals from the blood vessels and background tissue are eliminated in the dynamic contrast-enhanced image (shown in the yellow dotted box of Fig. 5f).

To quantify the performance of the *in vivo* dynamic contrast-enhanced imaging, we display multiple line profiles of the signals, extracted from the 3D images containing both the tumour and the blood vessels (positions of the lines shown in white triangles of Supplementary Fig. 7a–c); the signal intensities along these lines are normalized to the peak intensity for further analysis. Supplementary Fig. 7d,e shows one of these representative line profiles. By comparing the signals from the tumour region before and after CW laser irradiation, the Hoberman enhancement is 1.78±0.5 times, and the resulting contrast increases by 5.3 fold (see Methods, Data and statistical analysis section).

## Discussion

Dynamic contrast-enhanced photoacoustic imaging using the photothermal stimuli-responsive nanoconstructs highlights several interesting characteristics. First, we develop an approach to enhance photoacoustic signals with a controlled nanoparticle aggregation. More importantly, we show that the photoacoustic enhancement can be stimulated via modulated laser heating using stimuli-responsive polymer as nanoparticle carrier. Further, we experimentally demonstrate contrast-enhanced imaging both *ex vivo* and *in vivo* with the dynamic contrast-enhanced photoacoustic imaging approach. We show that the unwanted background signals such as signals from blood in molecular imaging can be suppressed using this approach. Overall, the photoacoustic signal enhancement from PNIPAM-assisted nanoparticle clustering and background suppression from dynamic behaviour of PNIPAM-nanoparticle nanoconstructs jointly contribute to more than one order of magnitude improvement in the imaging contrast (see Methods, photoacoustic/ultrasound imaging and signal characterization section). Our results have shown great potentials in high contrast imaging while reducing the required dosage of contrast agents. Additionally, the contrast enhancement strategy can separate signals produced by contrast agents from unwanted background signals with a single-wavelength illumination in a single scan, thus eliminating the burden of spectral mixing/un-mixing for background-free photoacoustic molecular imaging.

## Methods

### PNIPAM and PNIPAM-AM nanogel syntheses

Nanogels of Poly(n-isopropylacrylamide) (PNIPAM) and poly(n-isopropylmethacrylamide-acrylamide) (PNIPAM-AM), respectively, are prepared via free radical precipitation polymerization^36^. An aqueous solution (90 ml) containing monomer, N-isopropylacrylamide (1.39 × 10^−2^ M), and cross-linker, N-N′-methylene-bis-acrylamide (3.78 mM) is placed in a 250 ml three-neck flask with a reflux condenser. The solution is degassed under argon purge with mechanical stirring at 300 r.p.m. for 15 min and then heated to 70 °C in a water bath for 2 h. Then 10 ml of potassium persulfate solution (5 wt%) are injected into the flask via a needle. Following synthesis, the PNIPAM nanogels are purified several times by centrifugation (20,000 *g*, 30 min at 20 °C, Eppendorf 5804R) and re-dispersed in ultrafiltrated deionized water (18 MΩ cm, Thermo Scientific Barnstead Diamond water purification systems). The procedure to produce PNIPAM-AM nanogel is similar to the above protocol with replacement of the 10 mol% of monomer from N-isopropylacrylamide by N-isopropylmethacrylamide^23^ and with a reduced reaction time from 2 to 1 h. For PNIPAM-AM core PEG shell nanogel, 5 extra wt% of poly(ethylene glycol) methyl ether methacrylate (Mn: 950) in 5 ml of water is purged by argon then added to the reaction via needle^35^. The thickness of the polyethylene glycol (PEG) layer is about 10±5 nm based on this procedure. The reaction mixture is kept at 70 °C for 30 min. The same purification procedure as in PNIPAM is used to wash out the unreacted monomers and initiators.

### Copper sulfide nanoparticle synthesis

Copper sulfide (CuS) NSs are synthesized by reacting copper(II) chloride (CuCl_2_) with sodium sulfide (Na_2_S)^28^. Around 1 ml of Na_2_S_(aq)_ solution (1 M) is added to 1 l of an aqueous solution of CuCl_2_ (1 mM) and sodium citrate (0.68 mM), stirring at room temperature for 5 min. The resultant brown-colour reaction solution is then heated to 90 °C on a hotplate and stirred for another 15 min until a dark green solution is obtained. To introduce a PEG coating, 1 mg of NH_2_-PEG-SH (5 KDa) is added into the citrate CuS NS solution. The solution is stirred at room temperature overnight.

### Gold nanorod synthesis

Gold nanorods (AuNRs) are synthesized by seed-mediated growth^37^. Briefly, 5 ml of cetyl trimethylammonium bromide (CTAB) aqueous solution (0.20 M) is first mixed with 5 ml of chloroauric acid (HAuCl_4_) aqueous solution (0.5 mM). Then, 0.60 ml of ice-cold sodium borohydride solution (0.01 M) is added to the mixture and vigorously stirred for 2 min at 25 °C, which results in the formation of a brownish-yellow seed solution. The growth solution is made by adding 0.15–0.2 ml silver nitrate AgNO_3(aq)_ solution (4 mM) and then 5 ml of HAuCl_4(aq)_ (1 mM) solution to 5 ml of CTAB_(aq)_ (0.20 M) solution, under gentle mixing, followed by 70 μl of ascorbic acid (0.0788 M) solution. To grow nanorods, 12 μl of the seed solution is added to the growth solution at 27–30 °C under gentle stirring for 30 s. The transparency of the solution changes to burgundy red within 10–20 min. The solution is then aged for another 12 h at 27–30 °C, before being centrifuged at 18,000 *g* for 45 min, twice. The collected nanorods are re-dispersed in ultrafiltrated deionized water.

### PNIPAM-AuNR and PNIPAM-AM-AuNR nanoconstruct syntheses

To produce PNIPAM-AuNR and PNIPAM-AM-AuNR nanoconstructs, AuNRs are *in situ* grown inside the matrix of the nanogels directly by seed mediated growth method. In this method, the gold seeds are first selectively grown inside the nanogels. Particularly, 2.2 ml of nanogel solution (2.3 wt% in 0.14 M CTAB aqueous solution) is mixed with 25 μl of HAuCl_4(aq)_ solution (1 mM). Then, 0.60 ml of ice-cold sodium borohydride solution (0.01 M) is added to the mixture and vigorously stirred for 2 min at 25 °C, and the solution is incubated for 2 h. While waiting for the seed growth reaction, AuNR growth solution is prepared. The solution is constituted of 9.5 ml of CTAB solution (0.1 M), 0.4 ml of HAuCl_4(aq)_ solution (0.4 ml), 10–60 μl of AgNO_3(aq)_ solution (10 mM) and 64 μl of ascorbic acid solution (0.1 M). Then, 0.1 ml of seed solution is added to the growth solution to grow AuNRs from the seeds. To increase the concentration of the AuNRs loaded per nanogel particle, the concentration of the HAuCl_4(aq)_ solution in the seed solution is increased from 1 mM, to 5 mM, then to 15 mM, respectively. The solution is aged for another 12 h at 27–30 °C, before being centrifuged at 5,000 *g* for 45 min, twice. The collected nanoconstructs are resuspended in ultrafiltrated deionized water. To prepare ICG conjugated PNIPAM-AM-AuNR nanoconstructs, 100 μl of indocyanine green n-succinimidyl ester (1.2 × 10^−2^ M) in phosphate buffer saline (PBS, pH 7.4) is mixed with 100 μl of thiol-PEG_5_-amine (2.4 × 10^−3^ M) in PBS. The reaction is kept at room temperature for 2 h then quenched with 200 μl of Tris buffer (1 M, pH 7.5). The ICG-PEG_5_-thiol solution is mixed with 1 ml of PNIPAM-AM-AuNR aqueous solution (OD=3.3 at 1 cm optical path) overnight at room temperature. The ICG conjugated nanoconstruct is then purified twice with centrifuge at 5,000 *g* for 45 min. The collected nanoconstructs are re-dispersed in PBS, then its OD is adjusted to 20 (1 cm optical path).

### PNIPAM-CuS nanoconstruct synthesis

A mixture of CuS NSs and 1 ml of the PNIPAM nanogels (2.0 wt% solid) synthesized by the previously described protocol are combined in a tube. The mixture is put into a shaker overnight at room temperature and then washed twice by centrifugation at 3,000 *g* for 10 min. The loading concentration is controlled by varying the concentration ratio of CuS NSs to PNIPAM.

### Characterization of nanoconstructs

The optical properties of the nanoparticles and the nanoconstructs are optically characterized by UV-Vis extinction spectroscopy. Extinction spectra are collected from a 100 μl nanorod suspension in a temperature controlled 96-well microliter plate reader (BioTek Synergy HT) at room temperature. To investigate thermally induced optical property changes of nanoconstructs, the nanoconstruct solutions are heated to 32, 37, 42 and 50 °C, respectively, with plate reader. After reaching the target temperature, the temperature is maintained for 1 min before acquiring spectra. The size of the nanoconstructs is measured with temperature controlled dynamic light scattering (DLS) using Zetasizer (Malvern). In these DLS measurements, the nanoconstruct solutions are heated to a preset temperature and maintained for one minute before measurements. The number of the nanoconstructs is measured with nanoparticle tracking analysis system (NanoSight NS300, Malvern) at 25 °C.

The shape and morphology changes of the nanoconstructs are monitored with TEM imaging. To visualize the de-swelling state of the nanoconstructs, both the nanoconstruct solutions and copper-Formvar TEM grids are heated to 42 °C on a hotplate; then the heated solutions are drop-casted on the TEM grids, and air dried on the hotplate. The prepared grids are imaged using the TEM mode of a Hitachi S-5500 FESEM equipped with a field emission electron source operated at 30 kV.

### Photoacoustic/ultrasound imaging and signal characterization

To investigate the photoacoustic performance of PNIPAM-AuNR nanoconstructs, their photoacoustic signals are characterized using a commercial photoacoustic imaging system; the results are compared with the photoacoustic signals generated by their pure AuNR counterparts. Before imaging, the extinction of PNIPAM-AuNR and AuNR solutions are matched at OD=3.33 (for 1 cm optical path). An ultrasound micro-imaging system (Vevo 2100/LAZR, VisualSonics, Inc.) with a 20 MHz array ultrasound transducer (LZ250, VisualSonics, Inc.) is used to capture both ultrasound and photoacoustic signals. An optical fibre bundle for light delivery is integrated with the ultrasound transducer. The tissue-mimicking tube phantoms are made by first mixing each solution in 1:1 volume ratio with phenol-free Matrigel (Corning), a gelatinous protein that mimics the complex extracellular matrix found in many tissues. Then, both mixtures are injected separately into 10-cm-long thin wall polyethylene tubes (1 mm outer diameter and 0.6 mm inner diameter) and mounted on a plastic scaffold. Two phantoms consisting tubes filled with contrast media are prepared: one phantom is used for imaging and another—for signal calibration. The imaging phantom is shown in Fig. 2a. The phantom for signal calibration is constructed with two parallel tubes about 10 mm apart and placed in a water tank during imaging. The water inlet and outlet of the tank are connected to a temperature-controlled water circulator to maintain a constant and uniform temperature during imaging. The transducer with the fibre bundle is mounted on top of the phantom with a fixed distance of 1 cm from the transducer to the tube inclusions, which is aligned perpendicularly with respect to the imaging plane of the transducer. Seven-nanosecond laser pulses produced by a wavelength-tunable optical parametric oscillator (Premiscan, GWU, Inc.) are used to excite photoacoustic signals. The 780 nm laser with fluence at the output of the fibre bundle is adjusted to 5 mJ cm^−2^ by absorptive neutral density filters to prevent thermal damage of the AuNRs^38^. During the imaging, the water temperature is initially set at 13 °C and gradually increased to 42 °C by increments of 2 °C, then it is gradually cooled to 15 °C. The water temperature stays at each temperature for at least 1 min before the photoacoustic images are recorded to allow sufficient thermal diffusion in samples. At each temperature, three-dimensional (3D) photoacoustic images of the phantom are collected by stacking 300 B-mode images scanned along the direction perpendicular to the imaging plane. The photoacoustic performance of PNIPAM-CuS comparing to pure CuS NSs (Fig. 3b) is measured with a similar manner except a customized photoacoustic/ultrasound imaging system is used. This customized imaging system consists of an optical parametric oscillator (OPO) pumped by a nanosecond Nd:YAG laser (OPOTEK, 7 ns, 10 Hz), a needle hydrophone with central frequency of 20 MHz (Precision Acoustics), and a computer controlled data acquisition system. Same fluence (5 mJ cm^−2^) of 1064, nm laser beam with 1 cm^2^ spot size is used in these measurements.

### Animal studies

All animal experiments are performed in compliance with the Guidelines for the Care and Use of Research Animals established by the Stanford University Animal Studies Committee, under the protocol APLAC-13024. Healthy male nu/nu mice at age 6 weeks are used in this study. A prostate cancer in mouse model is developed by subcutaneously injecting 100 μl of 5 × 10^6^ LNCaP cells mixing with 1:1 volume ratio of growth factor reduced Matrigel into the right flank of each mouse. The tumour is allowed to grow to about 100 mm^3^ before imaging. Before imaging, mice are anesthetized with 2% isoflurane at 2 l min^−1^ of oxygen flow and confirmed with tail pinch. Around 50 μl of nanoconstructs/phosphate-buffered saline solution (3 × 10^10^ nanoconstructs with ∼1 × 10^12^ AuNRs, OD=20 in one centimetre optical path) is injected to the mice through tail veins.

### *Ex vivo* imaging

In *ex vivo* photoacoustic/ultrasound imaging, a 12 weeks old Nu/Nu mouse is euthanized by carbon dioxide asphyxiation followed by cervical dislocation to confirm death. Samples of 50 μl of CuS and PNIPAM-CuS aqueous solution (OD=6 for 1 cm optical path) mixed with Matrigel (Corning Inc., New York, NY, USA) at 1:1 volume ratio are implanted subcutaneously into the flank of the mouse. The two implants are 5 mm apart. The same ultrasound micro-imaging system (Vevo 2100, VisualSonics, Inc.) with a 20 MHz array ultrasound transducer (LZ250, VisualSonics, Inc.) is used for imaging. The mouse is immersed in a 25 °C water bath and the transducer is scanned along the dorsal direction of the mouse with a scanning volume 23 × 20 × 25.8 mm^3^ and a step size of 89 μm. To acquire the dynamic contrast-enhanced image, a 1.6 W cm^−2^ continuous wave (CW) Nd:YAG laser at 1,064 nm with a spot size of 1.1 cm^2^ is used for irradiating the imaging area where the samples are injected. During photoacoustic/ultrasound imaging acquisition, the CW laser is controlled by a function generator and synchronized with the imaging system to turn on and off by a square waveform with a 60 s period. The more than an order of magnitude imaging contrast enhancement is calculated based on measurements presented in Fig. 4. The contrast enhancement is defined as the signal to noise (here the dominated noise is from intrinsic photoacoustic signal) ratio (SNR) in the dynamic contrast-enhanced image with the PNIPAM-CuS nanoconstructs divided by the SNR in the photoacoustic image with the CuS NSs only while the laser is off. This ratio is adjusted for the differences in the overall photoacoustic magnitude. A clustering enhancement factor of 7 for the nanoconstructs in the *ex vivo* experiment is used for this estimation. The enhancement factor is obtained from the particle calibration in the *in vitro* experiment in Fig. 3 (concentration 3) and can be also calculated from measurements shown in Fig. 4e.

### *In vivo* imaging

For *in vivo* imaging, photoacoustic imaging and dynamic contrast-enhanced imaging are recorded using the same method as in the *ex vivo* imaging. In this study, a 780 nm nanosecond laser with fluence of 5 mJ cm^−2^ at the output of the fibre bundle is used and a CW solid state diode laser at 808 nm with a spot size of 1.1 cm^2^ are used for imaging. The 23 × 20 × 10.2 mm^3^ volume is mechanically scanned with a step size of 89 μm.

### Optical imaging

Epi-fluorescent images are recorded using IVIS spectrum imaging system (Perkin-Elmer). Both photoacoustic/ultrasound and fluorescent images are recorded before the particle injection. Fluorescent images are acquired at 10 min, 1 h, 2 h, 4 h and 24 h after injecting the particles.

### Data and statistical analysis

For data analysis, MATLAB is used to read the image data acquired with Vevo imaging system. The ultrasound images are shown in dB scale, and the photoacoustic and dynamic contrast-enhanced images are shown in linear scale. The two-dimensional (2D) images shown in Fig. 2c are the maximum intensity projections of the 3D volumetric images to the plane of lateral axis and elevational axis of the transducer. The photoacoustic signal intensities in Figs 2f,g and 3 are calculated by first summing the PA intensities inside each tube from each frame, defined by the ultrasound B-mode image, and then taking the average of 300 frames. In Fig. 4c,g, the contrast is defined as the ratio of the highest signal from the PNIPAM-CuS region to that from the CuS region. Specifically, the contrast of the photoacoustic image (CW laser off) is 4.4±0.8, and the contrast of the dynamic contrast-enhanced image is 30.8±1.2. The enhancement of the contrast—the ratio between these two contrasts, is 6.9 fold. In Fig. 5, the contrast is defined as the ratio of the maximum signal from the tumour region to that from the blood vessel region. The contrast enhancement is calculated based on the signals in Supplementary Fig. 7d,e, highlighted within the dotted box. The contrast of the PA image (CW laser off) is 2.7±0.6, and the contrast of the dynamic contrast-enhanced image is 14.3±1.9. The enhancement of the contrast is 5.3 fold. The error bars are the s.e. of the mean of the 300 s.d.'s. Data plot, average, s.d. and s.e. of the mean are computed in OriginLab 2009.

Three-dimensional ultrasound and photoacoustic images in Figs 4b–d and 5c–f are reconstructed using Amira software. To produce dynamic contrast-enhanced image, each frame of 2D photoacoustic images is first thresholded above the imaging system noise floor using a custom algorithm written in MATLAB. The noise floor of the imaging system is determined from a photoacoustic image of water. The noise fluctuation of each frame is compensated by taking the average of the photoacoustic signals over a 1 × 1 mm^2^ square area in the empty space between the imaging objects and the transducer of each frame. Each frame of dynamic contrast-enhanced image is produced by taking the differential image of photoacoustic images of each cycle (CW laser on and CW laser off) and then by averaging three cycles. The dynamic range of the dynamic contrast-enhanced image is then scaled back to the same scale of the photoacoustic image (CW laser off) to compare the signal to noise ratio.

### Data availability

The data that support the findings of this study are available from the corresponding author on request.

## Additional information

**How to cite this article:** Chen, Y.-S. *et al*. Dynamic contrast-enhanced photoacoustic imaging using photothermal stimuli-responsive composite nanomodulators. *Nat. Commun.* **8,** 15782 doi: 10.1038/ncomms15782 (2017).

**Publisher's note:** Springer Nature remains neutral with regard to jurisdictional claims in published maps and institutional affiliations.

## Supplementary Material

Supplementary Information Supplementary Figures, Supplementary Note 1 and Supplementary References

Supplementary Movie 1 A real-time recording of photoacoustic images with repeated on-off cycling of the external stimulus laser
