# Supplementary material for: Dynamic contrast-enhanced photoacoustic imaging using photothermal stimuli-responsive composite nanomodulators
Source: Nat Commun. 2017 Jun 8;8:15782. doi: 10.1038/ncomms15782 (PMC5472754; doi:10.1038/ncomms15782)
Supplement: Supplementary Information — Supplementary Figures, Supplementary Note 1 and Supplementary References [file ncomms15782-s1.pdf]

Supplementary figures

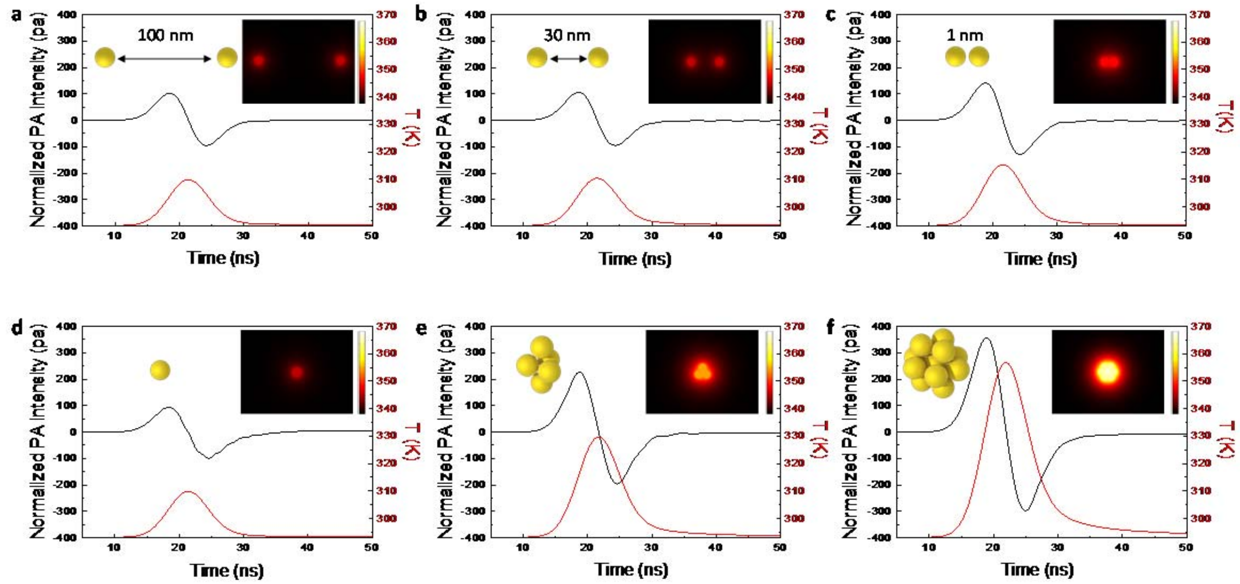

Supplementary Figure 1| Numerical simulations of photoacoustic signal enhancement due to aggregation. Photoacoustic intensities per particle and temperature rises as a function of time for gold nanospheres with distance varying from **a**, 100 nm, **b**, 30 nm and **c**, 1 nm. Photoacoustic intensities per particle and temperatures for clusters of gold nanospheres with **d**, 1, **e**, 5 and **f**, 13 particles. The particle distance for cluster simulations is kept at 1 nm. The insets show the two-dimensional temperature distribution at the peak temperature (t~21.5 nsec).

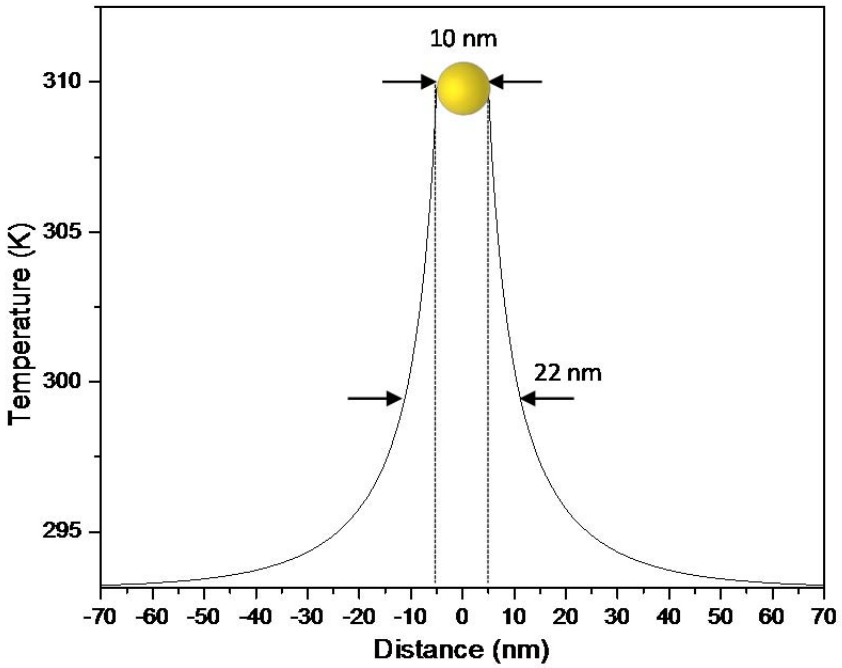

Supplementary Figure 2| Numerical calculation of temperature profile as a function of space. Heat induced by laser irradiation dissipates out from 10 nm gold nanosphere to raise the temperature of its surrounding water. The temperature decays quickly, and the decay length is characterized by the radius of the thermal diffusion, a distance where the amplitude of the temperature falls to its 1/e from the surface of the nanoparticle. In this case the thermal diffusion radius is 6 nm.

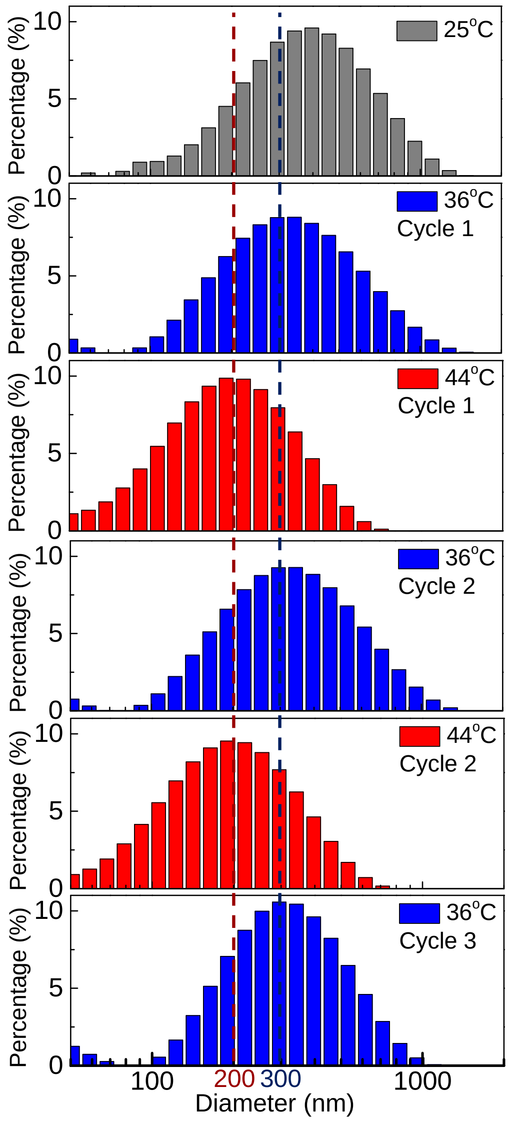

Supplementary Figure 3| Dynamic light scattering (DLS) measurements of PNIPAM-AM-AuNRs nanoconstruct indicate its LCST is above 37 ˚C. DLS measurements of PNIPAM-AM-AuNRs nanoconstruct at 25˚C (grey), 37˚C (blue), and 44˚C (red). The results show that the size of the nanoconstructs slightly reduces from 350 nm at room temperature to 320 nm at 37˚C. In multiple heating cycles, the size of the nanoconstructs can be repeatedly changed, following the modulation of temperature below and above LCST. The average size of nanoconstructs reduces from 320 nm at 37˚C (below LCST) to 195 nm at 44˚C (above LCST). The dotted lines indicate the mean values of the diameters of the nanoconstructs below and above LCST.

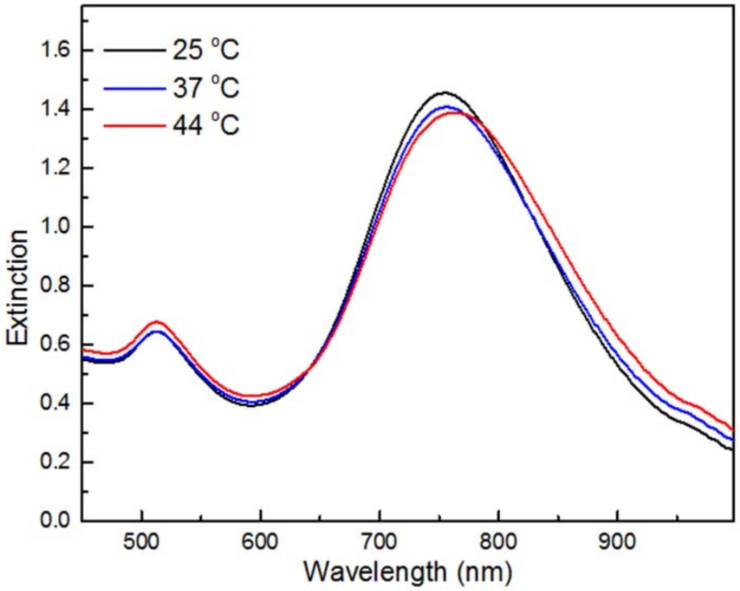

Supplementary Figure 4| UV-Vis spectra of PNIPAM-AM-AuNR nanoconstructs at 25˚C (black curve), 37˚C (blue curve), and 44˚C (red curve) are recorded, featuring the temperature below and above the LCST of PNIPAM. Above the LCST temperature (>37 ˚C), the aggregation of PNIPAM-AM-AuNR nanoconstructs increases light scattering, resulting in the raise of spectrum at a shorter wavelength. Although the DLS measurements (Figure S3) indicate the size of the PNIPAM-AM-AuNRs is slightly reduced, no obvious aggregation is observed from UV-Vis spectrum.

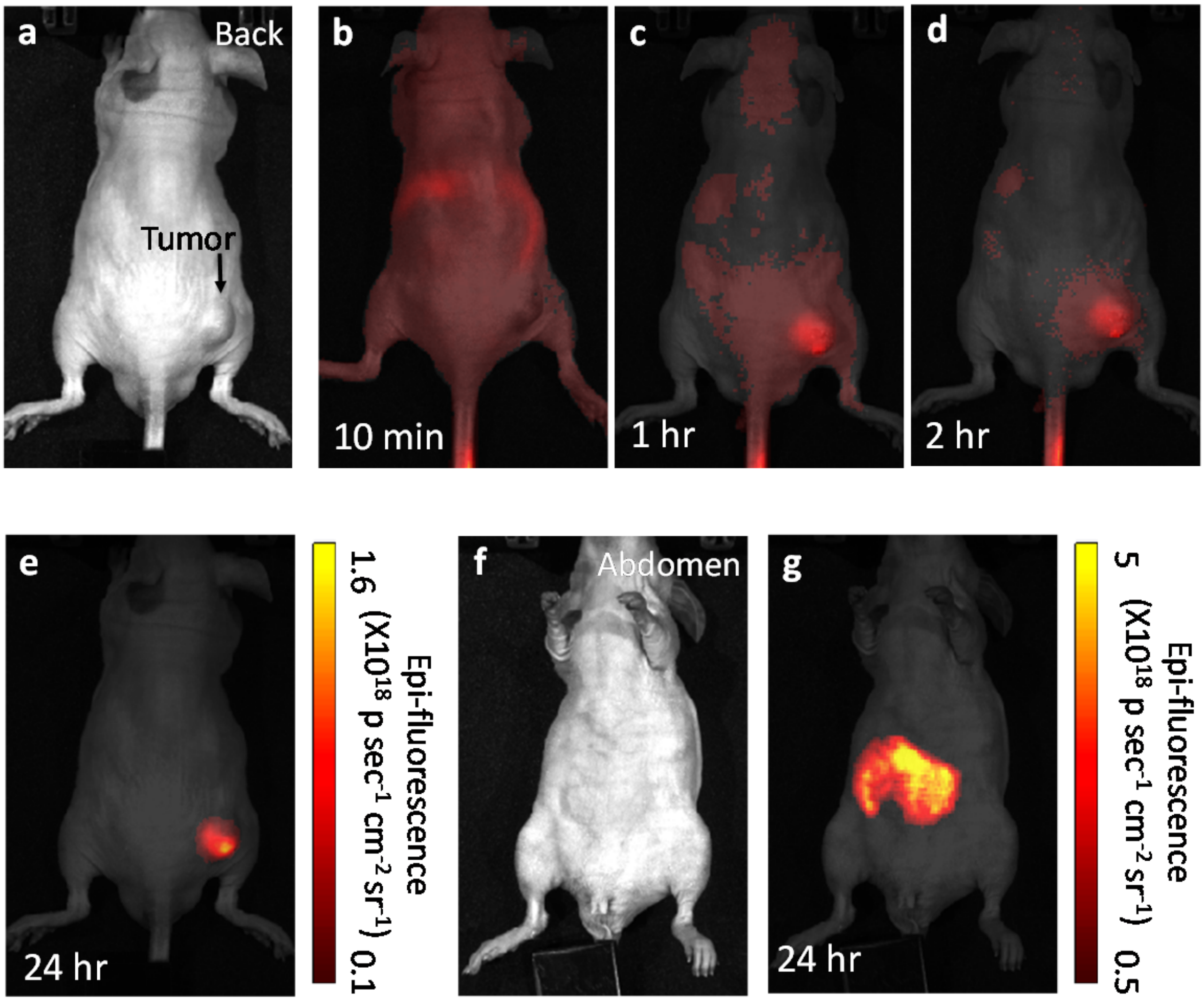

Supplementary Figure 5| *In vivo* epi-fluorescent imaging. a. Photographic image of the mouse from the back, showing the tumor on the right rear leg. Fluorescent imaging of the mouse **b**. 10 minutes, **c**. one hour, **d**. two hours, and **e**. 24 hours after tail vein injection of the nanoparticles. **e**. Abdomen photographic image of the mouse. **g**. Epi-fluorescent image of the abdomen of the mouse, showing accumulation of the nanoconstruct in the liver.

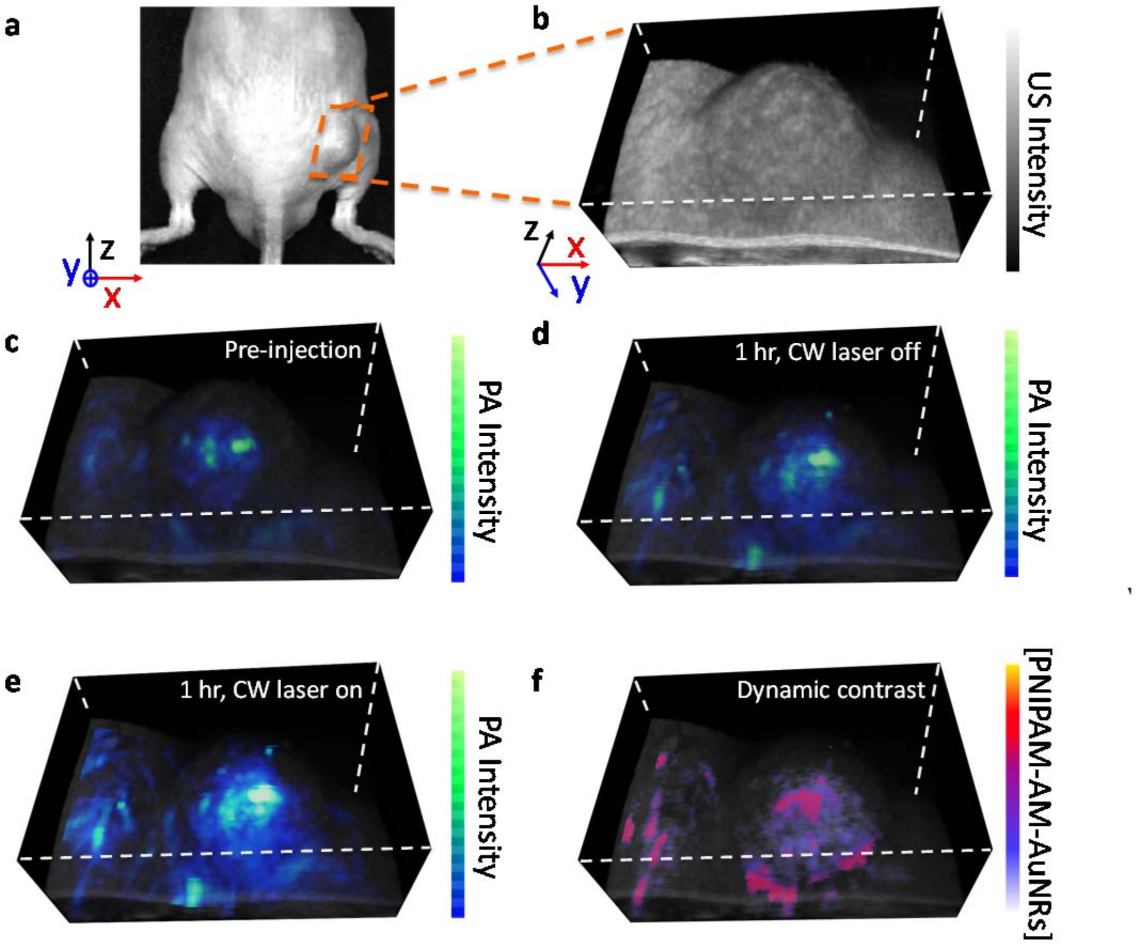

Supplementary Figure 6| *In vivo* photoacoustic imaging of a tumor bearing mouse at 1 hour after injection of nanoconstructs. a, Photograph of a subcutaneous prostate tumor in the right thigh of a mouse. **b.** Ultrasound image of the tumor for image co-registration. **c.** Photoacoustic image before nanoparticle injection shows the tumor, and blood vessels near the tumor. **d**, After 1 hour of nanoparticle injection, photoacoustic intensity of both tumor and blood vessels increases due to the high concentration of the nanoconstruct residue in the blood. **e**, When the CW laser is on, the photoacoustic intensity is further enhanced due to the de-swelling of the nanoconstructs. **f**, Dynamic contrast-enhanced photoacoustic image obtained by subtracting the photoacoustic signals under CW laser-off from CW laser-on, removing the photoacoustic signals from blood and revealing the region with only the PNIPAM-AM-AuNRs. The image volume in **b-f** is 14.9 mm × 5.9 mm × 10.2 mm.

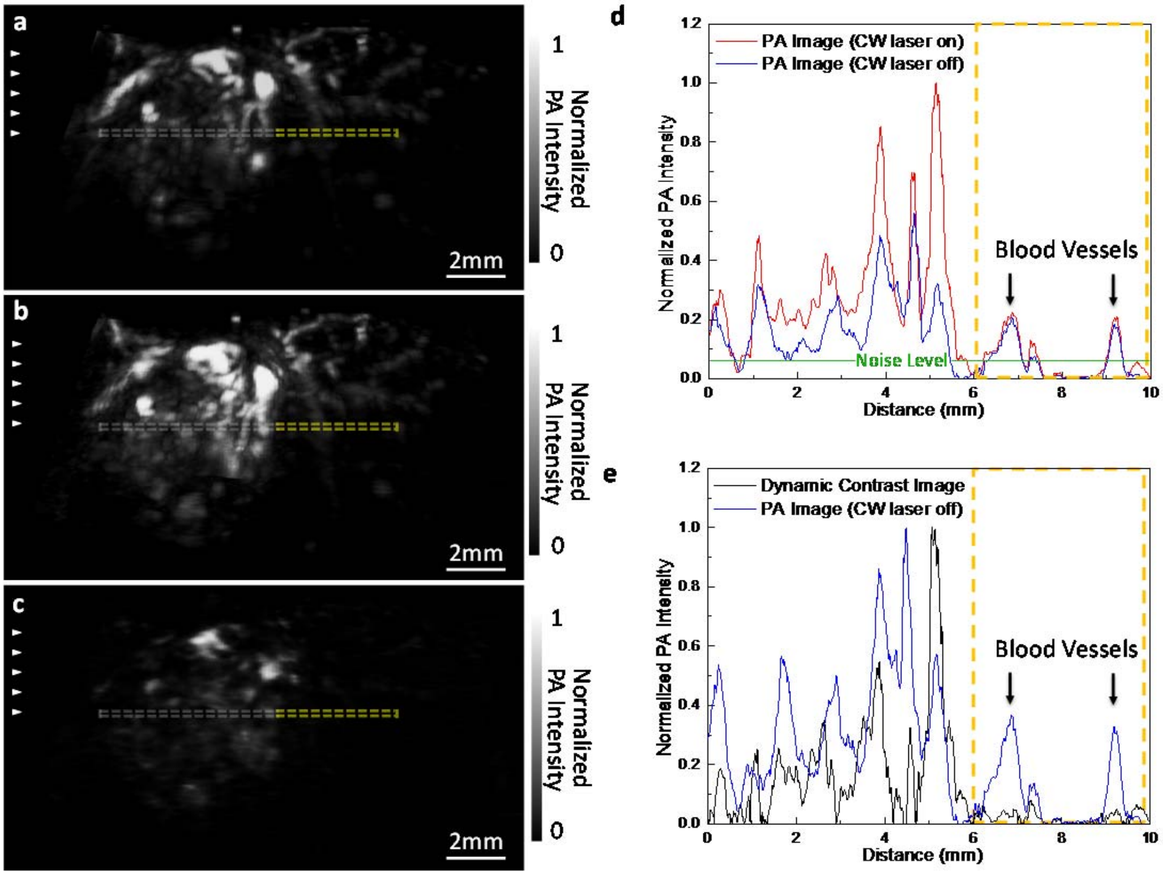

Supplementary Figure 7| *In vivo* photoacoustic and dynamic contrast-enhanced imaging of the mouse after 24 hours of injection. Normalized photoacoustic imaging of the mouse tumor region with PNIPAM-AM-AuNR nanoconstructs at 25˚C with **a.** laser off and **b.** laser on. The photoacoustic intensity in both image are normalized to maximum intensity of the image in panel (b)**. c**. Dynamic contrast-enhanced photoacoustic imaging by subtracting the photoacoustic images in panel (a) from panel (b). The image is then rescaled to match the highest intensity of the image in panel (a)**. d**. Averaged photoacoustic intensity line profiles extracted from the 5 pixel locations, indicated by the dashed box in panels (a) and (b), respectively. The green line indicates the electronic noise level of the imaging system. The dashed yellow box indicates the region with blood vessels seen in Figure 5. **e.** Averaged line profiles of the photoacoustic intensities extracted from the dynamic contrast-enhanced photoacoustic image in panel (c), from the same locations where the line-profiles of the static photoacoustic images are extracted in panels (a) and (b). The line profile from the laser off photoacoustic images is reproduced from panel (d), renormalized to reveal the lower noise level from the blood vessels in the dynamic contrast-enhanced imaging.

Supplementary Note 1. Numerical Simulations

Numerical simulations of PA intensities and temperature profiles as a function of time are conducted for gold nanospheres using commercially available software based on finite element method (COMSOL). The simulation incorporates a heat transfer model to calibrate temperature rise and a partial differential equation math model to calibrate the photoacoustic intensities. The photoacoustic intensity is recorded at a fixed point that is 300 nm away from the center of the nanosphere or clusters. The absorption cross-section of gold spheres is assumed to be 6×10^{-17} m^2 in the calculation based on Ref. 1. All nanospheres have a diameter of 10 nm, and their frequency-dependent permittivity is obtained from Ref. 2. Temporal profile of the laser pulse is assumed to be Gaussian with a full width half maximum of 7 nanoseconds.

The two dominant parameters important in describing random distribution of the particle aggregation are: 1) the average distance between particles, and 2) the number of particles aggregated in a fixed separation. We start from the first case by considering the simplest aggregation—two gold nanospheres. Our UV-vis spectroscopy measurement indicates that the nanoparticle aggregation affects optical absorption negligibly. Therefore, to focus on thermodynamic effect, we assume that optical properties of each gold sphere do not change as they are brought together. Here in our simulation, we use gold nanospheres with an optical absorption cross-section of 6×10^{-17} m^2.[1] **Supplementary Figure 1** shows that the photoacoustic intensity increases by 52% when the distance between two particles is reduced from 100 nm (**Supplementary Figure 1a**) to 1 nm (**Supplementary Figure 1c**). At the same time, this aggregation also affects temperature elevation within this nano-system by 1.8%.

Heat diffuses from the surface of the nanoparticles and decays exponentially, where the decay

length is characterized by the radius of the thermal diffusion, a distance at which the temperature falls to its 1/e from nanoparticle surface (~6 nm in this case, **Supplementary Figure 2**). When the two nanoparticles are more than 12 nm apart, the enhancement effect becomes negligible (**Supplementary Figure 1a, b**). Although the total energy absorbed by particles is the same in our calculation, a smaller particle separation increases the temperature in the gap and causes the photoacoustic signals to increase, because the photoacoustic signal intensity is directly related to the thermal flux.

In addition to the effect caused by the separation distance, we also investigate effect caused by the number of particle aggregation in a single cluster. When the nanosphere density is increased from 1 to 5, and to 13 with a fixed particle separation of 1 nm (**Supplementary Figure 1d-f**), both temperature and photoacoustic intensity are boosted dramatically: temperature is increased by 15.2% for 13 particles, whereas photoacoustic intensity per particle is increased by 285%. The increased numbers of nanoparticles more efficiently accumulate the heat within their gaps; because the photoacoustic signal intensity is directly related to the rate of temperature variation, the aggregation creates temperature "hotspots", resulting in a higher photoacoustic intensity. This indicates that if a nanoparticle cluster aggregation can be controlled by external stimuli, it will be an effective photoacoustic stimuli-responsive contrast agent.

Supplementary References

1. Liu, X., Atwater, M., Wang, J. & Huo, Q. Extinction coefficient of gold nanoparticles with different sizes and different capping ligands. *Colloids Surf., B* **58**, 3-7, (2007).
2. Johnson, P. B. & Christy, R. W. Optical constants of noble metals. *Phys. Rev. B* **6**, 4370-4379, (1972).
